# The importance of self-care and contextual factors: A process evaluation of a recovery intervention for new nurses

**DOI:** 10.1016/j.ijnsa.2026.100485

**Published:** 2026-01-07

**Authors:** Majken Epstein, Marie Söderström, Ann Rudman, Philip Tucker, Anna Dahlgren

**Affiliations:** a Department of Clinical Neuroscience, Karolinska Institutet, Solna, Sweden; b Department of Psychology, Stockholm University, Sweden; c Department of Health and Welfare, Dalarna University, Falun, Sweden; d School of Psychology, Swansea University, Swansea, United Kingdom

**Keywords:** Nurses, Occupational health, Primary prevention, Qualitative research, Recovery, Shift work schedule, Sleep

## Abstract

**Background:**

Newly graduated nurses often face demanding working conditions including high workload, stress, and irregular working hours. During the first years of practice, burnout symptoms are common. Recovery, including sleep, can be seen as a key protective factor in the associations between stress, shift work and negative health outcomes. Previously, a proactive, group-based intervention (recovery programme) for new nurses, promoting individual strategies for recovery, decreased burnout and fatigue symptoms post-intervention and showed preventive effects on somatic symptoms over time. To optimise the implementation and outcomes of an intervention, it is important to understand its mechanisms of impact (i.e., *how* it produces change) as well as to identify contextual factors influencing its implementation.

**Objective:**

To deepen the understanding of the recovery programme’s mechanisms of impact and to explore how its implementation, including participants’ opportunities for recovery and the feasibility of recovery strategies, was influenced by the context.

**Design:**

Qualitative descriptive design.

**Participants and setting:**

Twelve nurses (nine women) who had participated in the intervention at four Swedish hospitals, between 12 and 25 months (*M* = 19) after participation.

**Methods:**

Semi-structured individual telephone interviews were conducted and analysed using thematic analysis.

**Results:**

The programme’s proposed mechanisms of impact, including increased knowledge about sleep, enhanced motivation for behavioural change, and the use of recovery strategies, were confirmed. Motivation to apply recovery strategies was supported by a shift in mind-set regarding the importance of self-care; to improve readability; and follow-up on the behavioural change process during sessions. Contextual factors influencing recovery opportunities and the feasibility of strategies were related to both the work context and the individual. These factors included demanding schedules, extended and disrupted working hours, workload, opportunities for recovery at work, social norms, the organisation of work procedures, private life circumstances, and the deprioritisation of personal recovery needs. Booster sessions and reminders were suggested to facilitate the continued use of recovery strategies after the programme.

**Conclusions:**

When supporting nurses in developing individual recovery strategies, it is important to provide opportunities to share experiences with other new nurses and to follow-up on their behavioural change process. Importantly, several organisational factors should also be considered. Organisations should work systematically with the planning and management of working hours that promote recovery, create opportunities for recovery during work shifts, organise work procedures with recovery in mind, and continuously monitor and manage employees’ stress and fatigue symptoms. Together, such efforts could promote a social norm that supports recovery.

**Social media abstract:**

Nurses’ recovery can be supported through knowledge, individual strategies, follow-up, group reflections and organisational adaptations

**What is already known**

• Starting work as a new nurse is stressful, and symptoms of burnout are common.
• Insufficient recovery is a key mechanism linking long-term stress to poor health.

Alt-text: Unlabelled box

**What this paper adds**

• A changed mind-set towards self-care promotes the use of recovery strategies.
• A changed mind-set towards self-care promotes the use of recovery strategies.
• Work hours, workload, and social norms determine feasibility.
• Peer sharing and follow-up facilitate behavioural changes to improve recovery.

Alt-text: Unlabelled box

## 1 Background

Entering working life as a newly graduated nurse is a period characterised by high stress (Jarden et al., 2021) and has been described as a transition or reality shock (Kramer et al., 2013). Burnout symptoms, stemming from prolonged stress exposure, are prevalent among new nurses (Edwards-Maddox, 2023; Jarden et al., 2021). Early career burnout symptoms may have long-term consequences for nurses’ health (Rudman et al., 2020) and pose a risk to quality of care and patient safety (Jun et al., 2021). Burnout has further been linked to nurses’ intentions to leave their jobs (Dall’Ora et al., 2020), thereby threatening adequate hospital staffing. Accordingly, the World Health Organisation recently identified an urgent need to safeguard the mental health and well-being of nurses (World Health Organization, 2025).

Insufficient recovery has been suggested as an explanatory mechanism linking stress exposure to long-term health impairments (Geurts and Sonnentag, 2006). Recovery can be defined as the process of psychophysiological unwinding after effort, during which mental and physiological resources are replenished (Zijlstra et al., 2014). Sleep can be regarded as an essential source of recovery, facilitating the restoration of physiological systems (Åkerstedt and Nilsson, 2003) as well as psychological functions, including emotional regulation (Tomaso et al., 2021) and cognitive functioning (Lowe et al., 2017). In addition to sleep, recovery includes behaviours and activities during wake time that help to unwind, such as resting and relaxing, as well as more active pursuits contributing to restoring energy resources, like socialising, hobbies, and physical exercise (Sonnentag et al., 2017). In relation to work, psychological detachment from work-related thoughts during time off has been identified as one important aspect of the recovery experience (Sonnentag et al., 2022). Intervention studies show that supporting daily recovery behaviours in individuals with stress-related symptoms can reduce perceived stress, exhaustion, burnout, depression, and anxiety (Almén et al., 2020; Lindsäter et al., 2018). Moreover, a recent study evaluating a preventive stress intervention for new nurses identified the promotion of participants’ engagement in recovery behaviours as a valuable component (Rudman et al., 2024).

For many nurses, entering working life marks the beginning of shift work, which may compromise opportunities for both sleep (Harris et al., 2024) and other recovery activities. Nurses report a decline in sleep quality throughout their first years at work (Hasson and Gustavsson, 2010). Restricted sleep in itself contributes to fatigue and cognitive impairment (Lowe et al., 2017), thereby jeopardising patient safety. Combined with high work demands and difficulties to psychologically detach from work during time off, insufficient sleep may increase the risk of clinical burnout (Söderström et al., 2012). At the start of their careers, nurses may lack effective strategies for managing sleep and fatigue (Epstein et al., 2020). Moreover, during high-stress periods, when the need for recovery increases, recovery processes are often impaired or deprioritised, a phenomenon referred to as the ‘recovery paradox’ (Sonnentag, 2018). Accordingly, to safeguard the long-term health and work sustainability of nurses, as well as patient safety, it is essential to support new nurses in developing effective strategies for sleep and other forms of recovery.

### 1.1 A recovery programme for new nurses

Based on the above and data from our investigation of new nurses’ challenges with sleep and fatigue (Epstein et al., 2020), we developed a proactive, group-administered intervention (recovery programme) for new nurses. The recovery programme, which is described in more detail in Dahlgren et al. (2022), focuses on strategies promoting recovery to mitigate the negative impacts of work stress and shift work. It is based on techniques from cognitive behavioural therapy and consists of three manual-based group sessions covering the themes: 1) unwinding from stress; 2) promoting sleep in accordance with the principals of homeostatic and circadian sleep regulation; and 3) managing fatigue by increasing recovery behaviours. During sessions, psychoeducation is interspersed with follow-up on the participants’ behavioural change processes, group discussions, reflections and exercises. Between sessions, participants are encouraged to try strategies or make behavioural changes aimed at enhancing recovery, tailored to their individual situation, goals and values. Group leaders use approaches and communication skills inspired by cognitive behavioural therapy and motivational interviewing, such as open-ended questions, affirmations and reflections, to increase participants’ motivation for behavioural change and positively reinforce their efforts (Miller and Rollnick, 2012; Rummel et al., 2012). Functional behavioural analysis (Rummel et al., 2012) is also used as a tool to explore the consequences of habitual behaviours in stressful situations and to identify alternative responses.

A variety of strategies are presented in the programme, such as mindfulness, relaxation techniques, and different sleep-supporting behaviours, e.g., adjusting bedtimes to build up enough sleep pressure or exposure to daylight at optimal times of day. Other strategies include routines for leaving work (to enhance detachment from thoughts of work and unwinding during leisure), unwinding before bedtime, and practising daily recovery behaviours to manage and prevent fatigue. As nurses may have opportunities to influence their schedules (Wynendaele et al., 2021), the programme also includes guidance on how to schedule working hours to promote recovery, including access to a web tool (http://www.artur.portin.com) for evaluating schedules in terms of sleep, fatigue and potential recovery strategies in relation to different shifts.

Effects of the programme were evaluated in a randomised controlled trial involving 207 nurses from eight Swedish hospitals, all in their first year in the profession (Dahlgren et al., 2022). The evaluation showed a preventive effect on somatic symptoms in the intervention group. Burnout and fatigue symptoms were reduced in the intervention group at post-measurement; however, these differences were not significant at the 6-month follow-up.

### 1.2 Process evaluation

To optimise the outcomes and further implementation of an intervention, it is considered important to conduct a process evaluation as a complement to the evaluation of its effectiveness (Moore et al., 2015). According to the UK’s Medical Research Council guidance (Skivington et al., 2021), key functions of a process evaluation are to investigate 1) *what* was implemented and *how*, (2) *how* the intervention produced change (‘mechanisms of impact’), and (3) how the *context* may have affected the implementation and outcomes. In the trial evaluating the effects of the recovery programme, researchers led all group sessions, offering clear insight into *what* was implemented and *how*. Approximately 73 % of the 99 participants in the intervention group attended either two or all three sessions, and a brief survey distributed during the study indicated that participants tried several of the programme’s strategies (Dahlgren et al., 2022).

**Fig. 1:**
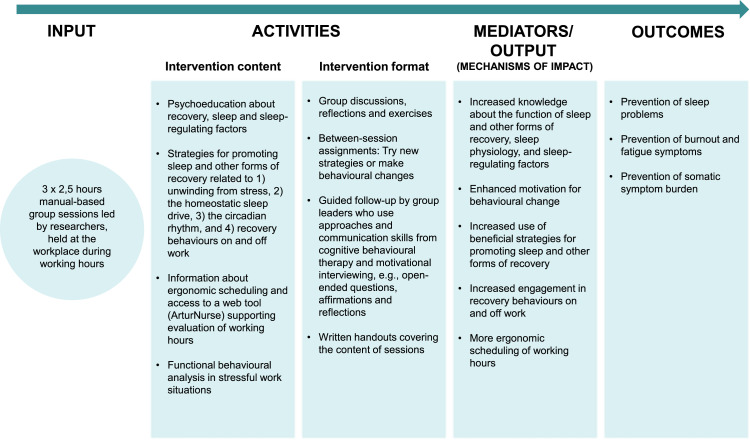
Logic model of the recovery programme.

However, the previous effect evaluation lacks a deeper understanding of the programme’s mechanisms of impact, that is, *how* it produced change. Proposed mechanisms include increasing participants’ knowledge of recovery and sleep; enhancing motivation for behavioural change; and supporting long-term use of strategies promoting sleep and other forms recovery, see Fig. 1. These mechanisms, however, could not be verified by the previous quantitative effect evaluation (Dahlgren et al., 2022). Nor could it identify which contextual factors that may have influenced nurses’ recovery opportunities and the feasibility of the strategies learned. A deeper understanding of such factors may clarify what contributed to the intervention’s positive effects, why effects on burnout and fatigue were not sustained at the 6-month follow-up, and which factors need to be addressed to optimise the implementation and outcomes of the intervention.

### 1.3 Aims

The aims of this process evaluation study were to 1) deepen understanding of the recovery programme’s mechanisms of impact (i.e., *how* it produced change) and 2) to explore if and how programme implementation, including participants’ opportunities for recovery and the feasibility of recovery strategies, was influenced by context.

## 2 Methods

### 2.1 Design and data collection

A qualitative descriptive design was adopted. Participants were recruited from both the intervention and control groups of the original intervention study, as both had received the intervention by the time of the interviews (the control group received it one year after the intervention group). The original intervention study was conducted over two years with different cohorts. For this interview study, only participants who had completed the programme one year earlier were initially invited. Due to a low response rate, the invitation was extended to include those who had completed the programme two years earlier. In total, email invitations were sent to 77 of the most recent participants of the intervention study, of whom 12 accepted.

Semi-structured individual telephone interviews were conducted between October 2019 and January 2020, 12 to 25 months (*M* = 19, *SD* = 6) after participation in the intervention. All interviews were conducted by author 3, a senior researcher (associate professor, registered nurse, female) with no prior personal contact with the participants. Before the interviews, participants were informed about the study’s aim. The interviews, which lasted 23–48 min (*M* = 36), were audio-recorded and transcribed verbatim before analysis. Toward the final interviews, the collected data were considered to provide sufficient richness to address the study’s aim (Braun and Clarke, 2021). Consequently, the number of participants who had responded to the interview request and completed interviews was deemed sufficient, and no further recruitment efforts were undertaken.

### 2.2 Participants

Participants (9 women, 3 men) were aged between 24 and 49 years (*M* = 31, *SD* = 8) and worked at four different hospitals at the time of the intervention. At the time of the interviews, most participants were still employed as hospital staff nurses, while a few had transitioned to nurse positions at primary health care centres.

### 2.3 The interview guide

The interview guide included open-ended questions aligned with the study’s aim. Minor revisions were made after the second interview to improve clarity for the interviewer. As these changes were only minor, the first and second interviews were included in the analysis. The interview questions addressed knowledge and insights gained; strategies the participants had used (with reminders of strategies provided); feasibility of strategies and barriers to using them or making behavioural changes; factors currently restricting sleep and other forms of recovery; changes to the scheduling process; elements missing from the programme; and opinions on the intervention format (e.g., group sessions, assignments). See Appendix A for the full interview guide.

### 2.4 Data analysis

Data were analysed using Braun & Clarke's (2022) six-phase thematic analysis. This method was chosen to facilitate the aim of identifying patterns, shared meanings, and variations across the dataset. In phase one, all interviews were read through independently by author 1 (MSc, licensed psychologist, female), author 2 (PhD, licensed psychologist, female) and author 5 (associate professor, female), to obtain an overview. This was a reflective phase including writing notes and reflections. In phase two, meaningful data relevant for the study’s aim were identified in the interviews and coded by author 1. Author 2 and author 5 coded 25 % of the interviews each, to confirm the process and ensure consensus in coding. In phase three, related codes were sorted and grouped, and the search for themes began with a discussion between authors 1, 2 and 5. In phase four, the interviews and codes were revised again by author 1, who discussed questions and uncertainties with authors 2 and 5. The groups of codes were reviewed and labelled as themes following a discussion between authors 1, 2 and 5. In phase five, the final themes were identified, described and named by authors 1, 2 and 5. In phase six, the content of the themes was formed, and the analysis was transcribed. Finally, author 3 checked the themes one last time against the raw data. The process was iterative, not strictly linear, and had overlapping steps.

### 2.5 Methodological considerations

To ensure trustworthiness in this study (Nowell et al., 2017), the following criteria were addressed: 1) Credibility: The analysis process included researcher triangulation. Multiple researchers were involved in coding, generation of themes and review of themes. The results were checked against the raw data to ensure referential adequacy. 2) Dependability: The entire analysis process is clearly described, step by step, to enable evaluation by the reader. 3) Confirmability: Quotations are provided throughout the manuscript to illustrate how findings were clearly derived from data. 4) Reflexivity: Authors from multiple disciplines were involved in the analyses, and their pre-understandings were frequently discussed throughout the analysis process. Author 1 is a licensed psychologist with experience in qualitative research on nurses’ sleep and fatigue, as well as in psychological treatments of sleep and stress-related problems. Author 2 is a licensed psychologist and an expert in sleep and stress related disorders and cognitive behavioural treatments. Author 3 is a registered nurse with extensive experience of qualitative research on similar topics, including nurses’ health and transition to practice. Author 5 is an expert in working hours and sleep, with prior experience from qualitative research on similar topics. Authors 1, 2 and 5 were involved in the original intervention study including recruitment, data collection, and the development and delivery of the intervention. Author 4 (associate professor), an expert in shift work research, contributed to manuscript drafting and critical revision. All authors have experience conducting research on nurses’ working environment and preventive interventions.

## 3 Results

**Fig. 2:**
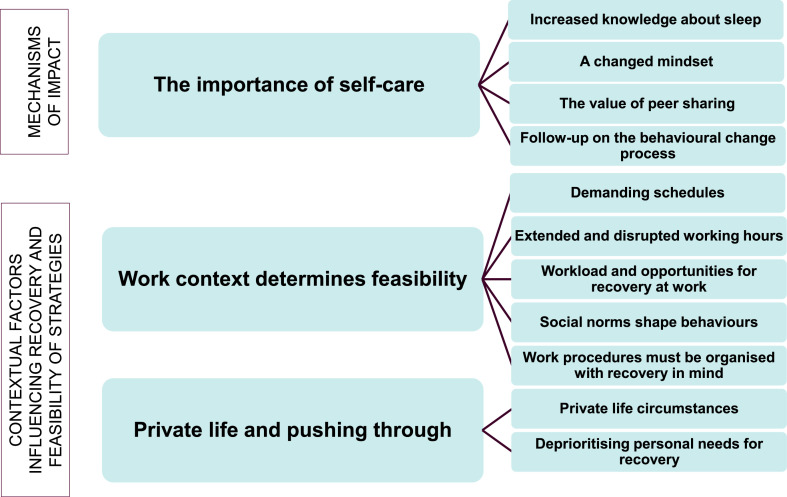
Coding tree illustrating main themes and sub-themes.

The qualitative analysis of the semi-structured interviews led to three main themes, each containing sub-themes illustrated in Fig. 2. The content of Theme 1 (*The importance of self-care*) addresses the primary aim of this study, which was to understand the mechanisms of impact of the recovery programme. Theme 2 (*Work context determines feasibility*) and Theme 3 (*Private life and pushing through*) address the study's secondary aim, which was to explore the contextual factors influencing recovery and feasibility of the recommended strategies.

The thematic content was discussed in relation to the various recovery strategies that respondents reported using after participating in the programme. These strategies reflected all the programme’s themes: unwinding from stress, homeostatic and circadian sleep regulation, recovery behaviours and ergonomic scheduling. While not all participants used every strategy, all but one – who considered the programme redundant due to already applying similar strategies – reported using at least one strategy learned during the programme. When asked, participants agreed that booster sessions of the programme would be helpful to enable repetition of the content and sustain motivation to prioritise recovery. Continuous reminders were also suggested to help maintain routines for using the strategies.

### 3.1 Theme 1: The importance of self-care

This theme highlights how the programme enhanced participants’ motivation to prioritise and make behavioural changes aligned with improved self-care. Increased knowledge about sleep, together with a shift in mindset that placed greater value on their own recovery, contributed to this motivation. Accordingly, participants’ approach to managing demands changed, allowing for a better balance between recovery and work engagement. Opportunities for peer sharing and continuous follow-up throughout the behavioural change process provided additional support for this approach.

#### 3.1.1 Increased knowledge about sleep

Participants felt that it was helpful to learn about sleep physiology and functions of sleep, factors regulating sleep and the interaction with working hours: *‘we were given guidance on how to think about sleep and how to structure it when working shifts, which I found very helpful’*(*Participant 2*) Also, by understanding and reflecting on how sleep affects daytime mood and functioning, it was easier to accept mood shifts during work after poor sleep: *‘if I feel I have a bad temper, I can reflect like “well I know my sleep was poor, it’s okay”’*(*Participant 10*). Having a better understanding of normal reactions to lack of sleep also contributed to decreased feelings of worry and less self-criticism.

#### 3.1.2 A changed mind-set

Some participants reported that, while they already had some of the knowledge taught in the programme, participation encouraged them to make the necessary behavioural changes*: ‘I think maybe it would have taken me a little longer to realise that I needed to take action and do something about it [if I had not participated]’*(*Participant 5*). Participants described a shift in their attitudes, or mind-sets, regarding recovery and self-care. The programme reminded them of the importance of prioritising their own health, sleep and recovery both during and outside of work: *‘I’m also a human who needs sleep, like my patients do (…) I didn’t think about it in that way before, I have always mostly thought about sleep as something that’s important for my patients (…) I value recovery much higher now than before’*(*Participant 7*). To prioritise recovery was also seen as beneficial for well-being, cognitive functioning and work performance. One nurse reflected:

‘I would never have sat down to rest [during the day] if no one had emphasised that it’s important. And at the end of the day, I think I’m a bit more focused, because I did that. So, it’s also about the whole mindset (…) you must somehow put yourself first (…) it’s a way of thinking (…) When you feel that it’s too much, say no, instead of pushing yourself through it, it will be better in the long run.’ (12)

Moreover, one participant reported that the programme helped her differentiate between factors which she could influence herself and factors that were the organisation’s responsibility, prompting her to initiate discussions with managers and colleagues.

#### 3.1.3 The value of peer sharing

Participants appreciated the small group format as it allowed them to reflect upon and share experiences of the programme content and recovery in relation to their work situation. They found it especially valuable to meet other new nurses in similar situations, as it helped alleviate the sense of being alone with their problems and enabled them to share beneficial coping strategies with one another:

‘It was very good to meet as a group, because everyone had different experiences and strategies to share (…) I felt like, it's not only me who has these problems, there are others too, and then I got to know how the others usually managed.’ (6)

Moreover, as the participants had no prior experience of being a nurse, they found it helpful to discuss with others what constituted reasonable and acceptable working conditions: ‘*when you are new in a profession, it’s difficult to know what’s reasonable (…) you rely a lot on your managers and your colleagues, and it becomes problematic when they expect you to work in a way that isn’t reasonable’* (7). It was noted that during the first professional year, nurses risk focusing solely on managing their work situation, neglecting their sleep, health and leisure. One nurse said: *‘there’s no other period in life where you sleep that bad, and are as stressed, as when you are a newly graduated nurse’*(*Participant 12*.

#### 3.1.4 Follow-up on the behavioural change process

The participants appreciated being encouraged to try strategies between group sessions and then meeting to follow-up on the behavioural change process. The guided follow-up by group leaders during the sessions increased motivation to try new strategies, encouraged reflection, and facilitated mutual problem-solving if something had been experienced as difficult. It was viewed positively that the group leaders continued to provide support throughout the programme, and ‘*that we got the chance to talk about it and get some advice’* (5).

### 3.2 Theme 2: Work context determines feasibility

This theme underlines how the work context often limited the participants’ opportunities for recovery behaviours, both *during* and *after* work hours. Barriers to recovery *during* work hours included a lack of structures for breaks, high workload, and social norms discouraging recovery. Recovery *after* work hours was described as largely affected by demanding schedules, disrupted working hours, and the way work was organised.

Participants emphasised that the recovery programme should not replace efforts to address organisational issues such as high workloads, staffing shortages, and demanding working hours. To manage work-related barriers to recovery, some participants had actively changed their work situation after attending the programme (e.g., changed workplace, switched to part-time or daytime work) due to high stress, unreasonable expectations from the employer (e.g., frequent overtime), poor recovery opportunities, sleep problems, or stress-related symptoms.

Moreover, some participants struggled to find time to attend the programme sessions, as participation during work hours was not permitted by the employer. Accordingly, it was suggested that the employer should be more actively involved in the programme implementation.

#### 3.2.1 Demanding schedules

Recovery behaviours, sleep, and the feasibility of strategies were hindered by demanding work schedules. One nurse said: *‘everyone should know what we have learned [in the programme] (…) but then it’s very difficult to apply [the strategies] because of the very irregular working hours’*(*Participant 3*). Problematic schedule characteristics included: mandatory quick returns; working every second weekend, unpredictable and fluctuating shift end times; only one day off between working periods; rotation between day, evening and night shifts; day-evening rotation within the same week; long shifts; and six consecutive working days (to allow longer time off in other weeks, e.g., during Christmas). Quick returns were especially challenging during high workload as stress after evening shifts hindered sleep, causing fatigue during the upcoming day shift.

Participants felt that the organisation should take greater responsibility for scheduling that promotes recovery. One participant evaluated her schedule using the web tool that was presented in the programme, which indicated that it was excessively fatiguing. She raised this with her employer and rescheduled her shifts, but the schedule was later adjusted again by the employer to meet ward needs, ending up being equally fatiguing as from the beginning.

However, there were also examples of workplaces making efforts to facilitate recovery. These included workplaces attempting to minimise the frequency of quick returns; delaying morning shift start times when employees had worked the evening before; and scheduling time for non-clinical work tasks (e.g., study time, scheduling) during the first hours of the evening shift, when the workload allowed.

#### 3.2.2 Extended and disrupted working hours

Having to work extra shifts or overtime, usually due to staffing shortages, hindered recovery and the feasibility of strategies. Working extra shifts caused sustained activation and disrupted strategies that had been established during the programme (e.g., unwinding before bedtime, taking regular meals): *‘that’s a great pity, during the programme I started establishing routines, but then by reasons related to work [working extra shifts], I’ve not been able to maintain them, they have been disrupted, so in the end I’ve not felt good anyway’*(*Participant 5*).

Another nurse explained:

‘Often, I feel like I can't recover (…) I work so much, and I work extra shifts, so I can’t do it. Like if I'm off for two days and they call from work, “well, it's someone sick, can you work this evening?” (…) and if I had planned certain activities, then I can't follow my plans because I have to help and work extra, when I’m trying to recover and rest.’ (6)

One participant reported having informed her employer that fatigue during overtime was affecting her performance. Nevertheless, the employer’s expectations of her performance level remained unchanged. Others described organisational adaptations related to extra shifts that supported recovery, for example, providing extra rest time or assigning less demanding tasks during double shifts.

#### 3.2.3 Workload and opportunities for recovery at work

High workload and stress were reported to hinder the use of recovery behaviours at work, often due to neglect or simply forgetting: *‘you don't remember it, you forget it after a while (…) it falls away when there’s a lot to do, so then you would need someone to remind you’* (9). During high workload, the ward atmosphere could change, making recovery even more difficult: ‘*it’s noticeable across the entire ward if it’s a stressful day, overall, it’s getting worse, a vicious circle is created’*(*Participant 12*).

Moreover, opportunities for engaging in recovery behaviours during the work shift, such as time for breaks, were not always present. One nurse explained:

‘It was difficult to change your own behaviour or your own conditions, since they were very dependent on the work environment. I felt that I had not very much control over it.’ (7)

It was also noted that stress at work made it more difficult to establish new habits, and work-induced fatigue after shifts reduced motivation to engage in recovery activities during free time (e.g., taking walks, exercising). One participant, comparing working as a nurse with a previous non-healthcare job, emphasised that healthcare organisations need to prioritise employee recovery more highly. It was seen as paradoxical that those working to improve others’ health were not given better opportunities to care for their own.

#### 3.2.4 Social norms shape behaviours

Social norms could hinder engagement in recovery behaviours. At one workplace, the prevailing ethos was to work a lot of overtime and to be *‘super-engaged’*(*Participant 7*), making it difficult to behave differently. Also, one participant felt that some more experienced colleagues *‘almost took pride in self-annihilation’*(*Participant 8*). It was also reported that taking care of one’s own recovery could be viewed as shameful in the healthcare sector: *‘you should stay busy with something all the time, otherwise you should be ashamed’*(*Participant 4*).

Sitting down for reflection with colleagues was not always socially accepted, and napping during night shifts was sometimes frowned upon, even when permitted by workload and systems designed to alert employees if needed.

Importantly, managers could influence workplace norms. One nurse, after changing workplace, described the positive impact of being respected for ‘*deserving*’ recovery: *‘now my managers ask how I feel and how I handle work (…) it makes a big difference’*(*Participant 7*).

#### 3.2.5 Work procedures must be organised with recovery in mind

Some work procedures were identified as hindering recovery. For example, the absence of handover routines well before the end of the shift caused stress during the final hour and impeded unwinding. One nurse reflected: *‘if you could re-structure the work procedures according to what’s best for the healthcare staff, I think you could do quite a lot (…) it’s so set in stone how a day is planned, how you do rounds and over-reporting (…) you could do a lot more for recovery’*(*Participant 8*).

Another barrier to recovery was the employer’s expectation that nurses retain patient knowledge across shifts by working quick returns, moving directly from evening to morning shifts, which allow little time for recovery. Conversely, one participant had initiated a change at her workplace after the programme. Before the change, nurses working quick returns were given priority to care for the same patients across both shifts, to facilitate continuity. Thus, quick returns, despite allowing little time for recovery, were rewarded by the organisation. After the change, nurses working two consecutive day or evening shifts were instead prioritised to care for the same patients during both shifts, encouraging shift combinations with better recovery opportunities.

### 3.3 Theme 3: Private life and pushing through

This theme highlights how aspects of private life influenced nurses’ opportunities for recovery and the feasibility of implementing recovery strategies. At times, private life circumstances posed a hindrance, and personal behavioural patterns of deprioritising one’s own recovery needs sometimes interfered with engagement in recovery strategies.

#### 3.3.1 Private life circumstances

Private life situations could hinder recovery and the practice of strategies from the programme. For example, family issues could prevent going to bed early before a morning shift. Worrying about private stressors could be a source of mental activation, making it difficult to fall asleep. Also, having family members with high support needs reduced available time for practising strategies from the programme.

#### 3.3.2 Deprioritising personal needs for recovery

Prioritising recovery strategies was sometimes hindered by personal behavioural patterns that involved pushing oneself through stressful situations. One example was when a lack of awareness of one’s own stress and fatigue symptoms, and consequent ignorance of their need for recovery, had led to clinical burnout:

‘I didn’t really realise how exhausted I was, so I couldn’t really put a stop to work either, because I was kind of used to pushing myself through stressful situations.’ (4)

Also, it was noted that high internal performance demands could hinder taking rest breaks. Another reason for pushing oneself and deprioritising recovery was a strong sense of responsibility for the work situation and schedules, often driven by loyalty to colleagues.

## 4 Discussion

The aim of this study was to deepen the understanding of the mechanisms of impact of a group-based recovery programme for new nurses, and to explore how programme implementation, including participants’ opportunities for recovery and the feasibility of recovery strategies, was influenced by context. The results show that participants used strategies learned in the programme, aligning with the intervention’s proposed mechanisms of impact – that is, enhanced motivation for and use of beneficial strategies that promote sleep and other forms of recovery. Strategies from all programme themes were used, supporting the value of offering a broad range of strategies. Increased knowledge supported behavioural changes, which also aligns with the proposed mechanisms. Importantly, the study shows that the work context crucially determined the extent to which recovery could be achieved and whether the programme content could be fully applied. Hence, the results indicate that the employer plays a critical role in creating opportunities for employees’ use of individual strategies for sleep and other forms of recovery. In summary, the study findings suggest that recovery among nurses should be supported at both individual and organisational levels.

Education and information alone are usually insufficient to change behaviours. This study therefore deepens the understanding of the factors that contributed to enhanced motivation for behavioural change, which are further discussed in the following sections.

Participants appreciated the group format, as it enabled the sharing of experiences and strategies with other new nurses, and valued the follow-up by group leaders on their behavioural changes. The group leaders’ efforts to verbally reinforce actions for behavioural change, along with other group members listening to, reflecting on and understanding participants’ experiences, may have served as social reinforcers for new behaviours and strategies. This accords with the concepts of positive reinforcement (Rummel et al., 2012) and operant conditioning (Skinner, 1963), which hold that experiencing desirable or rewarding consequences of a behaviour increases the likelihood of its continuation. Similarly, trying out strategies and experiencing them as helpful may have further strengthened participants’ motivation to continue using them.

Participants’ shift in mind-set regarding the importance of recovery and self-care likely also contributed to their motivation for change. This aligns with several health behaviour theories, which state that attitudes are one major determinant of performing health behaviours (Albarracín et al., 2024). According to self-determination theory, intrinsic motivation, rather than motivation driven by external rewards or social pressure, is key to sustaining behavioural change (Ryan and Deci, 2000). Participants’ changed mind-set may reflect a shift toward intrinsic motivation for recovery behaviours, as they began to personally endorse their value. It is plausible that the motivational interviewing techniques used by group leaders further supported such intrinsic motivation (Markland et al., 2005).

Moreover, according to self-determination theory, perceived competence and confidence in one’s ability to change are fundamental to intrinsic motivation. In this study, participants’ perceived competence was reflected in their descriptions of increased knowledge. The programme also helped distinguish between individual and organisational responsibilities, and identify both opportunities and limitations for change, which may have strengthened their sense of competence. Elements within the group sessions that followed up on participants’ use of strategies ensured that the programme provided not only skills and tools, but also support when barriers to change emerged – elements considered important for enhancing perceived competence (Ryan et al., 2008). Previous research has similarly shown that adding therapist support to self-help psychological sleep treatments can increase strategy adherence and improve outcomes (Kaldo et al., 2015).

Relatedness is another concept that, according to self-determination theory, supports intrinsic motivation (Ryan and Deci, 2000). In this study, sharing experiences and challenges with other nurses were appreciated aspects of the programme. These interactions likely enhanced participants’ sense of group relatedness, further contributing to their intrinsic motivation for change. Talking to others in similar situations, sharing feelings, and experiencing a sense of belonging were also identified as key elements in a previous intervention targeting work-related stress and burnout among healthcare workers (Peterson et al., 2008).

### 4.1 Barriers to engaging in recovery behaviours

Regardless of participants’ motivation for change, high workloads and poor working conditions were highlighted as barriers to engaging in recovery behaviours. Workload and stress could lead to forgetting or neglecting recovery, a result also evident in a recent preventive intervention study targeting stress-related ill-health among new nurses (Rudman et al., 2024). Such reports illustrate the recovery paradox, i.e., when the need for recovery increases, recovery tends to be impaired or deprioritised (Sonnentag, 2018), indicating that nurses may need organisational support to both enable and maintain recovery, especially during periods of high workload. A previous review identified potential organisational facilitators of nurses’ rest breaks, including designated and comfortable break areas, clear organisational break regulations, and managerial efforts such as establishing break schedules and providing reminders (Wendsche et al., 2017).

Another barrier to using recovery strategies was the presence of workplace social norms that discouraged recovery, specifically, norms that valued working much overtime, staying constantly busy, or avoiding breaks. The importance of the social context for new nurses’ coping strategies was also highlighted in a recent study, in which proactive strategies, such as seeking assistance or delegating tasks, were perceived as socially unacceptable and therefore not adopted (Rudman et al., 2024). These findings align with previous research suggesting that social norms are important determinants of health-promoting behaviours (Kim et al., 2019; Rivis and Sheeran, 2003).

The use of certain strategies was contingent on participants’ working time schedules, underscoring the need for organisations to provide schedules that allow for sufficient sleep and recovery between shifts. Moreover, the results showed that extended and disrupted working hours – such as overtime and extra shifts – interfered with strategies for recovery, for example by hindering the ability to unwind in the evenings and maintain regular eating habits. However, staffing shortages, combined with short-term absences and other unforeseen events, may pose a significant challenge for hospitals in maintaining 24/7 staffing without relying on nurses working overtime and extra shifts (Epstein et al., 2023). In this study, such additional work was associated with heightened fatigue at work and impaired performance, in line with previous research (Griffiths et al., 2014). This highlights the need for organisations to make adaptations both *during* work hours – e.g., by allowing more time for breaks or adjusting tasks to be less demanding – and *after* work hours*,* e.g., by delaying the start time of the morning shift if the employee has worked the evening before.

Making working hours a leadership priority will likely have positive long-term effects on employee health. A study found that managers of hospital wards with low levels of sick leave supported employees’ work-life balance by providing flexibility in working hours, tasks and vacations, when possible (Fällman et al., 2022). Such approaches may help employees to manage fatigue, sleep, and other forms of recovery, thereby contributing to better health. Similarly, we showed in an earlier study that active engagement in scheduling by first-line healthcare managers was a key factor in the promotion of sustainable working hours (Epstein et al., 2023). Examples of active engagement behaviours included fostering individual relationships with employees to understand personal circumstances, preferences, and shift tolerance; ensuring support in schedule planning; and addressing dissatisfaction related to working hours.

Several participants reported having changed their workplace or working hours (e.g., switching to part-time or daytime-only) after the programme. These changes were often prompted by ongoing stress symptoms, sleep problems, poor recovery or unreasonable employer expectations. This reflects participants’ view that the recovery programme could not fully mitigate organisational issues such as excessive workload, staffing shortages, and involuntary overtime. Also, one participant developed clinical burnout after the programme, highlighting that it is not suitable as treatment for incipient burnout. This was expected, given its brief and preventive nature. However, it underscores the need for organisations to complement preventive programs with monitoring of stress-related symptoms, to identify necessary adaptations that may help prevent the development of more serious problems.

The limitations of the individualised approach to occupational health have been previously highlighted by a meta-analysis showing that organisational interventions (e.g., shift pattern changes, reduced workload) were more effective in reducing burnout among physicians than individual interventions like psychoeducation and mindfulness (Panagioti et al., 2017). However, the strength of an individualised approach lies in its flexibility, as nurses may face various challenges related to stress and recovery (Epstein et al., 2020). An intervention combining an individualised approach (e.g., improving knowledge and skills) and an organisational approach may be most effective for preventing and managing stress-related ill-health (Awa et al., 2010). In the original intervention study (Dahlgren et al., 2022), we employed a train-the-trainer approach to enhance organisational knowledge and support integration of the recovery programme into the organisations’ structural support for employee recovery. Currently, some Swedish hospital organisations include the programme in their introductions for new nurses. Future studies should evaluate effects of the programme when delivered by the organisation, as well as facilitators and barriers to implementation.

Notably, organisation-directed interventions focusing on healthcare employee wellbeing appear to be less explored in the literature than individual-directed interventions (Cohen et al., 2023). However, one leadership-based approach to improving employee sleep is ‘sleep leadership’, consisting of behaviours through which leaders both encourage and enable healthy employee sleep (Adler et al., 2021). In military contexts, employees experiencing sleep leadership report better sleep quality and sleep quantity (Gunia et al., 2015) and reduced daytime sleep-related impairment (Sianoja et al., 2020). In line with the current study’s findings, a similar approach – expanded to include recovery behaviours during waking hours both in and outside of work – may complement the recovery programme and enhance its impact on recovery and health.

The finding from the intervention study, that reductions in burnout and fatigue symptoms post-intervention did not persist at the 6-month follow up (Dahlgren et al., 2022), may be partly explained by the difficulties with continuing using strategies during heightened stress and workload, as described by the participants. Also, the range of other work-related contextual factors identified as affecting recovery opportunities and the feasibility of strategies might have further reduced the programme’s long-term effects. In addition to addressing such work-related factors, participants noted that continuous reminders and/or booster sessions might facilitate continued strategy use. Previous studies have similarly shown that positive effects of interventions on work-related burnout can be enhanced by regular booster sessions following the programme (Awa et al., 2010). Another way to enhance the intervention’s effectiveness would be to encourage participants to create a personalised programme summary and plan for maintaining helpful strategies, as is common practise in cognitive behavioural therapy protocols (Barlow, 2021).

### 4.2 Practical implications

Based on the findings of the current study, several organisational adaptations are suggested to optimise recovery opportunities and enhance nurses' use of recovery strategies. Organisations could work actively to enable the use of recovery strategies during work hours, for example by providing time and structures for breaks. This may be particularly important during periods of high workload, when recovery paradoxically tends to be impaired and/or deprioritised precisely when it is most needed. Moreover, organisations should ensure that working hours support sleep and other forms of recovery. This may require leaders to take an active role in managing working hours, including offering flexibility in scheduling and ensuring that both planned and continuously adjusted working hours adhere to ergonomic scheduling principles. When employees are required to work overtime or extra shifts, their workload and tasks may need to be adjusted. For instance, they may benefit from longer breaks, less demanding tasks, or later shift start times if they worked the previous evening. Workplace routines and work tasks, e.g., handover procedures and rounds, may be reviewed to assess how their current structure influences recovery opportunities and how they might be optimised to promote recovery. If organisations and leaders place greater emphasis on recovery – both by highlighting its importance and by establishing the necessary conditions as outlined above – it is reasonable to expect that, over time, social norms within workgroups will shift towards greater acceptance and appreciation of recovery.

Importantly, organisational efforts to promote recovery should be complemented by monitoring employees’ stress-related symptoms and identifying potential adaptations that may help prevent the development of more serious health issues. To support new nurses in their specific challenges with recovery, opportunities should be provided to meet other nurses to facilitate the exchange of reflections and experiences regarding work-related challenges and recovery strategies.

### 4.3 Limitations

Some limitations of this study should be noted. Firstly, only 12 of approximately 110 participants that participated in the programme (when led by researchers) were interviewed, which may result in important perspectives or experiences being missed. Those who agreed to participate may also have been especially positive toward or interested in the intervention. However, the participants represented four (of eight) different hospitals, and both the variety and similarities in their experiences suggest that the study has captured different perspectives as well as important common experiences. Secondly, qualitative research is sensitive to interviewer and analyst bias. In this study, participants were not asked to provide feedback on transcriptions or the conclusions reached. Instead, credibility was ensured through researcher triangulation and careful comparison of findings with raw data. Additionally, the third author (who conducted all interviews and was involved in the analysis process) was not involved in the intervention study and thus had no prior relationship with the participants.

## 5 Conclusions

The recovery programme enhanced nurses’ motivation for behavioural change, i.e., use of recovery strategies, through increased knowledge and a shift in mind-set. Sharing experiences with other new nurses, along with follow-up on behavioural changes, were important elements contributing to engagement and motivation for change. Moreover, to facilitate the continued use of strategies after the programme, reminders and booster sessions may be helpful. However, contextual barriers can limit participants’ recovery opportunities and the feasibility of implementing recovery strategies. Therefore, to enable participants to fully apply the programme content and optimise its effects, organisations must work systematically with issues related to employee recovery. This should include planning and management of working hours to promote sleep and recovery, adapting workload or tasks during overtime and extra shifts, supporting engagement in recovery behaviours (particularly during high workload), considering how work procedures (e.g., handovers) affect recovery opportunities, and continuously monitoring and managing employees’ stress and fatigue symptoms. Together, such efforts could promote a social norm that supports recovery.

## Ethical considerations

This study was approved by the Ethics Review Board in Stockholm, Sweden (2016/1395–31/2) and followed the Declaration of Helsinki (World Medical Association, 2013). Participation was voluntary. All participants received written and oral information about the study and signed informed consent before the interview.

## Declaration of generative AI and AI-assisted technologies in the writing process

During the preparation of this work the authors used Microsoft 365 Copilot in order to improve language and readability. After using this tool, the authors reviewed and edited the content as needed and take full responsibility for the content of the publication.

## Funding

This study was funded by AFA Försäkring (180242).

## Data availability statement

The authors cannot share the raw data (interviews) since the participants have not agreed to that in the consent form.

## CRediT authorship contribution statement

**Majken Epstein:** Writing – review & editing, Writing – original draft, Formal analysis, Conceptualization. **Marie Söderström:** Writing – review & editing, Writing – original draft, Formal analysis, Conceptualization. **Ann Rudman:** Writing – review & editing, Investigation, Formal analysis, Conceptualization. **Philip Tucker:** Writing – review & editing, Conceptualization. **Anna Dahlgren:** Writing – review & editing, Writing – original draft, Project administration, Funding acquisition, Formal analysis, Conceptualization.

## Declaration of competing interest

The authors declare that they have no known competing financial interests or personal relationships that could have appeared to influence the work reported in this paper.

## Appendix Supplementary materials

Image, application 1
