# Supplementary material for: The importance of self-care and contextual factors: A process evaluation of a recovery intervention for new nurses
Source: Int J Nurs Stud Adv. 2026 Jan 7;10:100485. doi: 10.1016/j.ijnsa.2026.100485 (PMC12834931; doi:10.1016/j.ijnsa.2026.100485)
Supplement: Supplementary file 1 [file mmc1.docx]

| **Part 1: The recovery programme “Bädda för Kvalitet”** |
| --- |
| - Would you like to tell me about your overall experience of participating in the recovery programme Bädda för kvalitet? - The recovery programme included both an educational component and practical exercises involving different strategies. Regarding the educational content, what did you learn from the programme? - Which strategies or advice from the programme have you continued to use after its completion, and how has that changed over time?   - *Why those particular strategies?*   - *Why the change over time, if any?*   - *Have you used any of the strategies introduced in the programme?*   ***Interviewer gives examples of strategies included in the programme, if not mentioned by the respondent:***   - - *Unwinding bedtime routine*   - *Body scan/mindfulness, “closing the time window”*   - *Situation analysis: Situations, reactions, behaviours, short-term consequences and long-term goals for behavioural change. Observe own behaviours and situations.*   - *Routine for leaving work*   - *Personal goal for supporting sleep related to the homoeostatic and circadian processes*   - *Short relaxation technique*   - *“The energy wheel”*   - *Practicing recovery behaviours at work and at home* - Have there been any hinders to applying what was presented in the recovery programme and behavioural changes?   - Contextual factors? Has anything in your work or personal context made it difficult to implement behavioural changes? |
| **Part 2: Sleep, fatigue and recovery** |
| - How is your situation today, do you face any current challenges in achieving good sleep and recovery? - In the recovery programme, we discussed how sleep is affected by different work schedules. Have you changed your way of scheduling your shifts? - Did you use the ArturNurse webtool to evaluate your schedules?   - *If not used, why?* |
| **Part 3: Overall summary** |
| - Was there anything you felt was missing from the intervention programme? - Was there anything in the programme that was unclear or difficult to understand? - Was there anything you found particularly helpful? |
| **Part 4: Suggestions for improvement** |
| - Did you experience that the programme was delivered at an appropriate point in your nursing career? - How did you like meeting in a group setting? - What are your reflections on the homework assignments? - You received written materials for each group session. How did you like those? - Do you think it would have been helpful to have a booster session of the programme a while after? - What do you think the recovery programme could mean for newly graduated nurses? - To summarise, how would you describe your recovery today? Are you satisfied with it? |
